# Chronic Intermittent Hypoxia Exacerbates High-Fat Diet-Induced MASLD Through Lipid Metabolic Reprogramming, Impaired Antioxidant Defense, and NF-κB/NLRP3 Activation

**DOI:** 10.3390/biom16050751

**Published:** 2026-05-20

**Authors:** Lisong Ye, Jihang Le, Xiaofei Lei, Fujie Song, Yichan Guo, Jun Gao, Yuehua Liu

**Affiliations:** 1 Department of Orthodontics, Shanghai Stomatological Hospital & School of Stomatology, Fudan University, Shanghai 200001, China; lsye23@m.fudan.edu.cn (L.Y.); 22301010025@m.fudan.edu.cn (J.L.); 22301010073@m.fudan.edu.cn (X.L.); jef0512@foxmail.com (F.S.); 2 Shanghai Key Laboratory of Craniomaxillofacial Development and Diseases, Fudan University, Shanghai 200001, China; 22301010027@m.fudan.edu.cn

**Keywords:** chronic intermittent hypoxia, metabolic dysfunction-associated steatotic liver disease, lipid metabolic reprogramming, oxidative stress, Nrf2/GPX4, NF-κB/NLRP3

## Abstract

Chronic intermittent hypoxia (CIH), the cardinal pathophysiological feature of obstructive sleep apnea, is increasingly recognized as an important modifier of metabolic dysfunction-associated steatotic liver disease (MASLD), but the underlying mechanisms remain incompletely understood. In this study, male C57BL/6 mice were fed a standard diet or a high-fat diet (HFD) and exposed to normoxia or CIH for 8 weeks. Histological, ultrastructural, biochemical, transcriptomic, proteomic, and metabolomic analyses were integrated to characterize hepatic alterations induced by CIH under metabolic stress. CIH markedly aggravated HFD-induced liver injury, as evidenced by increased body fat, hepatomegaly, serum transaminases, steatosis, mitochondrial ultrastructural alterations, and inflammatory infiltration. Mechanistically, CIH promoted hepatic lipid metabolic reprogramming by suppressing the PPARα/CPT1A fatty acid β-oxidation axis while enhancing the SREBP-1c/FASN/PLIN2 lipogenic pathway, impaired the Nrf2/HO-1/SLC7A11/GPX4 antioxidant defense system, increased lipid peroxidation and iron accumulation, and activated NF-κB/NLRP3 signaling. These findings support a multifactorial model in which CIH functions as an additional hypoxic stressor that exacerbates HFD-induced MASLD-like liver injury through coordinated metabolic, oxidative, and inflammatory dysregulation.

## 1. Introduction

Metabolic dysfunction-associated steatotic liver disease (MASLD) has become the most prevalent chronic liver disease worldwide and is increasingly recognized as a major hepatic manifestation of systemic metabolic dysfunction [1,2]. Its pathological spectrum ranges from simple steatosis to metabolic dysfunction-associated steatohepatitis (MASH), fibrosis, cirrhosis, and even hepatocellular carcinoma [3]. Current concepts indicate that MASLD progression is not driven by a single insult, but by the coordinated action of multiple pathogenic factors, including nutritional overload, insulin resistance, lipotoxicity, oxidative stress, and chronic inflammation [4,5,6]. Within this framework, a high-fat diet (HFD)-induced metabolic burden is considered a major initiating factor, whereas additional systemic stressors may further promote the transition from simple steatosis to active liver injury [7].

Obstructive sleep apnea (OSA) is a common metabolic comorbidity that has attracted increasing attention in the context of MASLD [8]. The cardinal pathophysiological feature of OSA is chronic intermittent hypoxia (CIH), which is characterized by repetitive cycles of hypoxia and reoxygenation during sleep [9]. Accumulating clinical and epidemiological evidence indicates that OSA is associated with increased severity of steatosis, hepatocellular injury, and fibrosis, even after adjustment for obesity and other shared metabolic risk factors [10]. These findings suggest that CIH may act as an additional hypoxic stressor within a multifactorial disease model, thereby aggravating liver injury in metabolically vulnerable individuals [11]. However, despite the increasingly recognized association between OSA-related hypoxia and MASLD progression, the molecular basis underlying this interaction remains insufficiently defined [12].

Several mechanisms have been proposed to explain how CIH aggravates hepatic injury. Intermittent hypoxia can enhance systemic lipolysis and increase the influx of free fatty acids into the liver, thereby worsening hepatic lipid overload [13,14]. In parallel, repeated hypoxia–reoxygenation cycles resemble ischemia–reperfusion-like stress and promote excessive production of reactive oxygen species (ROS), mitochondrial stress, and redox imbalance [15]. These alterations may further contribute to hepatocellular injury and disease progression [16,17]. However, most previous studies have focused on isolated pathways or individual mediators, making it difficult to define the broader molecular basis by which CIH reshapes the metabolically stressed liver [18]. Therefore, a systematic approach is needed to clarify the key pathogenic programs through which CIH accelerates MASLD progression under conditions of pre-existing nutritional stress.

In the present study, we combined transcriptomic, proteomic, and metabolomic profiling with histological, ultrastructural, biochemical, and molecular validation to investigate how CIH accelerates HFD-induced MASLD. We aimed to characterize the hepatic molecular alterations induced by CIH in the setting of metabolic overload and to identify the key pathogenic pathways linking hypoxic stress to progressive liver injury. By delineating these coordinated changes, this study seeks to provide a more comprehensive understanding of how CIH promotes MASLD progression and to identify potential therapeutic targets for patients with concomitant OSA and metabolic liver disease.

## 2. Materials and Methods

### 2.1. Animals and Experimental Desig

Male specific pathogen-free C57BL/6 mice (6 weeks old, *n* = 32) were purchased from Shanghai SLAC Laboratory Animal Co., Ltd. (Shanghai, China). Mice were housed under standard specific pathogen-free conditions with a 12 h light/dark cycle and free access to food and water. After one week of acclimatization, mice were randomly assigned to four groups (*n* = 8 per group): Control, HFD, CIH, and HFD+CIH. The Control group received standard chow under normoxia; the HFD group received a high-fat diet under normoxia; the CIH group received standard chow under chronic intermittent hypoxia; and the HFD+CIH group received a high-fat diet combined with CIH exposure. The entire experimental protocol, including dietary intervention, CIH exposure, sample collection, and euthanasia, was reviewed and approved by the Animal Ethics Committee of Shanghai Jiao Tong University School of Medicine (Approval No. A2025178). All animal experiments were conducted in accordance with the institutional guidelines for the care and use of laboratory animals and the Guidelines for the Ethical Review of Laboratory Animal Welfare. The study was reported in accordance with the ARRIVE guidelines.

The primary outcomes of this study were hepatic steatosis and liver injury, assessed by liver histology, Oil Red O staining, liver weight, liver index, and serum transaminase levels. The secondary outcomes included targeted mechanistic readouts related to hepatic lipid metabolism, oxidative stress and lipid peroxidation, inflammatory responses, and hepatocellular ultrastructure, as well as exploratory transcriptomic, proteomic, and metabolomic profiles associated with CIH exposure under HFD conditions.

### 2.2. High-Fat Diet Feeding and Chronic Intermittent Hypoxia Exposure

Mice in the HFD and HFD+CIH groups were fed a purified high-fat diet (XTHF60, 60% kcal from fat; Xietong Pharmaceutical, Nanjing, China) for 8 weeks, whereas mice in the Control and CIH groups received standard chow. Chronic intermittent hypoxia was induced using a programmable hypoxia chamber system (OxyCycler C42, BioSpherix, Parish, NY, USA). The inspired oxygen concentration was decreased from 21% to 5 ± 1% over 150 s, maintained for 30 s, and then restored to 21 ± 1% over 120 s, yielding a 5 min cycle (approximately 12 cycles/h). Mice were exposed to CIH for 8 h/day for 8 consecutive weeks. Control and HFD mice were maintained in identical chambers under normoxic conditions.

### 2.3. Sample Collection

At the end of the intervention, mice were fasted for 12 h with free access to water. Before sacrifice, body composition was assessed in live mice using a quantitative magnetic resonance body composition analyzer (QMR06-090H) according to the manufacturer’s instructions, and fat mass was recorded as an indicator of adiposity. Mice were then anesthetized with 1% pentobarbital sodium, and blood samples were collected from the orbital venous plexus. After centrifugation, serum was collected and stored at −80 °C. Livers were rapidly excised, weighed, and processed for histological, biochemical, ultrastructural, molecular, and omics analyses. The liver index was calculated as liver weight/body weight × 100%. Samples for RNA, protein, and multi-omics analyses were snap-frozen in liquid nitrogen and stored at −80 °C until use. Image quantification and data analysis were performed blinded to group allocation.

### 2.4. Serum Biochemical Analysis

Serum ALT, AST, TG, TC, HDL-C, and LDL-C were measured using commercial kits (BIOBASE, Jinan, China) on an automated biochemical analyzer (BK-280, BIOBASE, Jinan, China) according to the manufacturers’ instructions. To reduce batch effects, all samples were analyzed as consistently as possible within the same batch. Each sample was measured twice, and the mean value was used for statistical analysis.

### 2.5. Histological Analysis

Liver tissues were fixed in 4% paraformaldehyde for 24 h, paraffin-embedded, and sectioned at 4 μm for hematoxylin and eosin staining. For Oil Red O staining, liver tissues were embedded in OCT compound and sectioned at 8 μm. H&E staining was used to evaluate hepatic histopathological changes, and Oil Red O staining was used to assess hepatic neutral lipid accumulation.

### 2.6. ROS Staining and Quantification

Frozen liver sections were subjected to ROS fluorescence staining using a DCFH-DA-based reactive oxygen species assay kit (Beyotime, Shanghai, China), followed by DAPI counterstaining. For quantification, three non-overlapping fields from the same anatomical region of each section were randomly selected at 200× magnification. Image acquisition parameters were kept identical across groups. ROS levels were quantified using ImageJ software (version 1.54g; National Institutes of Health, Bethesda, MD, USA) and expressed as mean fluorescence intensity (MFI).

### 2.7. Immunohistochemistry and Immunofluorescence

Paraffin-embedded liver sections were deparaffinized, rehydrated, and subjected to antigen retrieval. After blocking, sections were incubated overnight at 4 °C with the indicated primary antibodies. For immunohistochemistry, an HRP-based polymer secondary antibody system (Servicebio, Wuhan, China) and a DAB kit (Servicebio, Wuhan, China) were used for visualization, followed by hematoxylin counterstaining. For immunofluorescence, Alexa Fluor 488- or 594-conjugated secondary antibodies (Jackson ImmunoResearch, West Grove, PA, USA) were used, and nuclei were counterstained with DAPI (Abcam, Cambridge, UK). Images were acquired under identical settings, and positive staining area or fluorescence intensity was quantified using ImageJ.

### 2.8. Oxidative Stress Assays

Liver tissues were homogenized, and the supernatants were collected for assessment of oxidative stress-related indices. Hepatic SOD activity, GSH-Px activity, MDA content, and hepatic iron content were measured using commercial kits (Aidisheng, Nanjing, China), including the SOD assay kit (ADS-F-KY011), GPX assay kit (ADS-F-G003-48), MDA assay kit (ADS-F-YH002), and iron assay kit (ADS-F-D007), according to the manufacturers’ instructions. Parallel wells were set for each sample, and the mean value was used for analysis. Samples with a coefficient of variation >10% were re-tested. Results were normalized to tissue fresh weight.

### 2.9. ELISA

Serum TNF-α, IL-6, IL-1β, and IL-10 levels were determined using commercial ELISA kits (Jiangsu Jingmei, Yancheng, China), including the TNF-α kit (JM-02415M2), IL-6 kit (JM-02446M2), IL-1β kit (JM-02323M2), and IL-10 kit (JM-02459M2), according to the manufacturers’ protocols. Absorbance was read at 450 nm using a microplate reader (Infinite F50, Tecan, Männedorf, Switzerland). Each sample was assayed in duplicate, and the mean value was used for statistical analysis. Samples with a coefficient of variation >10% were re-tested. To reduce batch effects, the same analyte was measured in the same batch whenever possible.

### 2.10. Transmission Electron Microscopy

Fresh liver tissues were cut into approximately 1 mm^3^ pieces and immediately fixed for transmission electron microscopy. After post-fixation, dehydration, resin embedding, ultrathin sectioning, and heavy-metal staining, samples were observed under a transmission electron microscope (HT7800, Hitachi, Tokyo, Japan) to evaluate hepatocellular ultrastructure, including lipid droplets and representative mitochondrial morphology. For TEM-based quantification, lipid droplet accumulation was quantified as lipid droplet area percentage using ImageJ. The lipid droplet area percentage was calculated as the total lipid droplet area divided by the total TEM field area × 100%. Quantification was performed in a blinded manner.

### 2.11. Multi-Omics Analyses

Liver tissues from six mice per group were subjected to transcriptomic, proteomic, and untargeted metabolomic analyses by Hangzhou Lianchuan Biotechnology Co., Ltd. (Hangzhou, China). The primary omics comparison was HFD+CIH versus HFD, which was used to identify hepatic molecular alterations associated with CIH exposure under HFD-induced metabolic stress.

For transcriptomics, total RNA was extracted, quality-assessed, and sequenced on the Illumina NovaSeq X Plus platform (San Diego, CA, USA). After read filtering, genome mapping, and expression normalization, differentially expressed genes were identified using DESeq2 with thresholds of FDR < 0.05 and |fold change| ≥ 2. Multiple testing correction was performed using the Benjamini–Hochberg method. The list of differentially expressed genes and the corresponding GO, KEGG, and GO-based GSEA results are provided in Tables S2–S5. For proteomics, samples were analyzed using the Astral-Zoom-DIA workflow on a Thermo Vanquish Neo UHPLC system coupled to a Thermo Orbitrap Astral Zoom mass spectrometer. Protein identification was controlled at PSM FDR < 0.01 and protein FDR < 0.01. After abundance normalization and quality filtering, differentially expressed proteins were identified using thresholds of fold change > 1.2 or <0.83 and *p* < 0.05. The list of differentially expressed proteins and the corresponding KEGG enrichment results are provided in Tables S6 and S7. For untargeted metabolomics, liver extracts were analyzed by LC–MS. Pooled quality-control samples were used to monitor analytical stability. After peak detection, alignment, annotation, normalization, and quality filtering, differential metabolites were identified using thresholds of *p* < 0.05, fold change > 1.2, and VIP ≥ 1. The list of differential metabolites and the corresponding KEGG enrichment results are provided in Tables S8 and S9. The omics analyses were considered exploratory, and pathway-level findings were prioritized for interpretation only when supported by phenotypic, histological, biochemical, or targeted molecular validation data.

### 2.12. RT-qPCR

Total RNA was extracted from frozen liver tissues using the FastPure^®^ Cell/Tissue Total RNA Isolation Kit V2 (RC112, Vazyme, Nanjing, China). cDNA was synthesized using HiScript IV All-in-One Ultra RT SuperMix for qPCR (R433, Vazyme, Nanjing, China). Real-time quantitative PCR was performed using SupRealQ Purple Universal SYBR qPCR Master Mix (U+) (Q412, Vazyme, Nanjing, China) according to the manufacturers’ instructions. Each sample was analyzed in triplicate. Relative mRNA expression was calculated using the 2^−ΔΔCt^ method with Actb as the internal control. Primer sequences are listed in Table S1.

### 2.13. Western Blotting

Total protein was extracted from frozen liver tissues using pre-cooled RIPA lysis buffer containing protease and phosphatase inhibitors on ice. After centrifugation at 12,000 rpm for 10 min at 4 °C, the supernatants were collected and protein concentrations were determined using a BCA protein assay kit. Equal amounts of protein (30 μg per lane) were separated by SDS-PAGE and transferred onto 0.22 μm PVDF membranes (Millipore, Burlington, MA, USA). After blocking with 5% non-fat milk or 5% BSA, membranes were incubated overnight at 4 °C with primary antibodies against PPARα (Bioss, Beijing, China; bsm-55599R, 1:1000), CPT1A (Proteintech, Wuhan, China; 15184-1-AP, 1:5000), PLIN2 (Proteintech, Wuhan, China; 15294-1-AP, 1:2000), SREBP-1c (Proteintech, Wuhan, China; 14088-1-AP, 1:1000), FASN (Proteintech, Wuhan, China; 10624-2-AP, 1:5000), Nrf2 (Cell Signaling Technology, Danvers, MA, USA; 12721, 1:1000), HO-1 (Proteintech, Wuhan, China; 10701-1-AP, 1:5000), SLC7A11 (Cell Signaling Technology, Danvers, MA, USA; 98051, 1:1000), GPX4 (Abcam, Cambridge, UK; ab125066, 1:1000), NLRP3 (Cell Signaling Technology, 15101, 1:1000), IL-1β (Cell Signaling Technology, 12242, 1:1000), TNF-α (Proteintech, Danvers, MA, USA; 17590-1-AP, 1:500), NF-κB p65 (Cell Signaling Technology, Danvers, MA, USA; 8242, 1:1000), phospho-NF-κB p65 (Cell Signaling Technology, Danvers, MA, USA; 3033, 1:1000), and β-actin (Proteintech, Wuhan, China; 200536-1-AP, 1:4000), followed by HRP-conjugated secondary antibodies. Protein bands were visualized using an ECL detection reagent (Thermo Fisher Scientific, Waltham, MA, USA; catalog no. 34579) and quantified using ImageJ, with β-actin as the internal control.

### 2.14. Statistical Analysis

Data are presented as mean ± SD. Statistical analyses were performed using GraphPad Prism 10.4.1. Data distribution was examined before parametric testing. Comparisons among multiple groups were conducted using one-way ANOVA followed by Tukey’s multiple-comparison test. No data points were excluded unless a technical failure was identified. No missing values were present in the conventional biochemical, histological, qPCR, or Western blot datasets; therefore, no imputation was performed for these analyses. For proteomic and metabolomic datasets, undetected or unreliably quantified features were handled during the corresponding preprocessing and quality-filtering workflows. A *p* value < 0.05 was considered statistically significant.

## 3. Results

### 3.1. CIH Aggravates HFD-Induced Metabolic Dysfunction and Liver Injury

**Figure 1:**
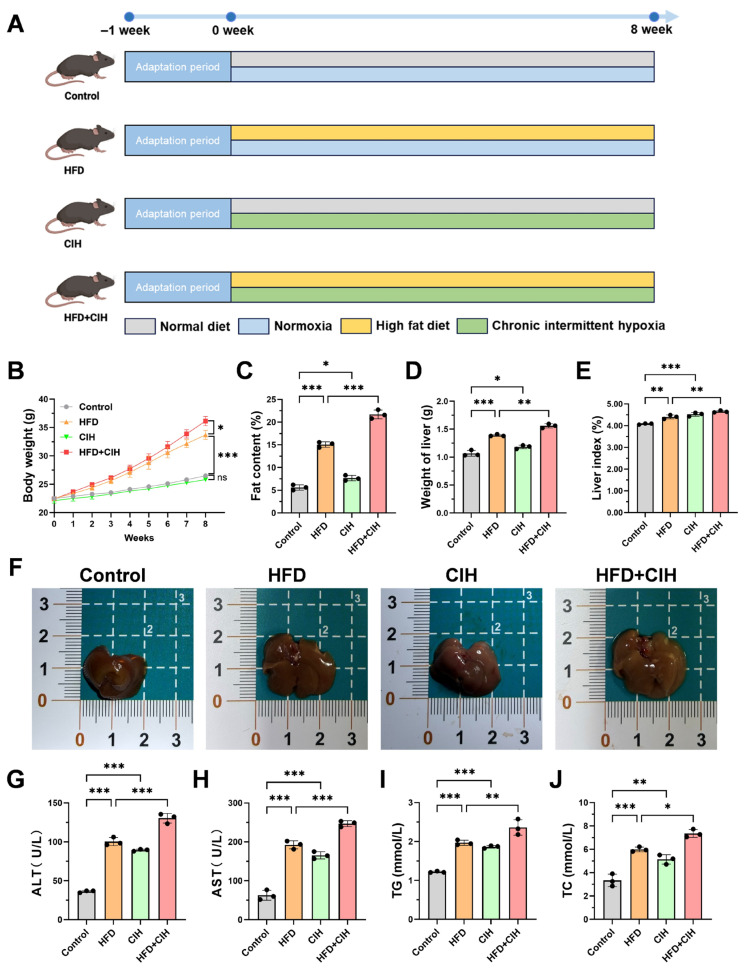
CIH aggravates HFD-induced metabolic dysfunction and liver injury in mice. (**A**) Schematic illustration of the experimental design. (**B**) Body weight changes during the intervention period. (**C**) Body fat content. (**D**) Liver weight. (**E**) Liver index, calculated as liver weight/body weight × 100%. (**F**) Representative gross morphology of livers from each group. (**G**–**J**) Serum biochemical parameters, including ALT (**G**), AST (**H**), TG (**I**), and TC (**J**). Data are presented as mean ± SD (*n* = 3). Statistical significance is indicated in the graphs: * *p* < 0.05, ** *p* < 0.01, *** *p* < 0.001.

To determine whether CIH aggravates metabolic dysfunction and liver injury under HFD-induced metabolic stress, general phenotypes, liver burden, and serum biochemical parameters were first examined in the four experimental groups (Figure 1A–J). As shown in Figure 1A, mice were fed standard chow or HFD and exposed to normoxia or CIH for 8 weeks. Body weight increased progressively in all groups during the intervention period; however, HFD-fed mice exhibited a greater increase than control mice. Notably, CIH further enhanced body weight gain in HFD-fed mice, resulting in a higher terminal body weight in the HFD+CIH group than in the HFD group (Figure 1B). A similar trend was observed for body fat content, which was markedly elevated by HFD feeding and further increased after CIH exposure (Figure 1C), indicating that CIH exacerbated obesity-related phenotypes under HFD conditions.

Consistent with these findings, liver burden was also aggravated by CIH under HFD conditions. Liver weight increased from 1.067 ± 0.052 g in the control group to 1.389 ± 0.018 g in the HFD group and further to 1.559 ± 0.038 g in the HFD+CIH group (Figure 1D). Likewise, the liver index increased from 4.08 ± 0.03% in the control group to 4.41 ± 0.09% in the HFD group and further to 4.66 ± 0.05% in the HFD+CIH group (Figure 1E). Gross examination showed that livers from HFD-fed mice displayed visible enlargement and discoloration, and these changes were more pronounced in the HFD+CIH group (Figure 1F), further supporting an aggravating effect of CIH on HFD-induced hepatic injury.

Serum biochemical indices were then analyzed to assess liver injury and systemic lipid abnormalities. HFD feeding markedly increased serum ALT and AST levels, and these elevations were further amplified by CIH exposure. Serum ALT increased from 36.3 ± 1.4 U/L in the control group to 100.3 ± 5.2 U/L in the HFD group and to 130.4 ± 6.2 U/L in the HFD+CIH group (Figure 1G). Similarly, serum AST increased from 63.0 ± 12.8 U/L in the control group to 192.5 ± 10.4 U/L in the HFD group and to 246.9 ± 7.6 U/L in the HFD+CIH group (Figure 1H). Serum TG and TC showed the same pattern. TG increased from 1.22 ± 0.02 mmol/L in the control group to 1.97 ± 0.07 mmol/L in the HFD group and to 2.36 ± 0.20 mmol/L in the HFD+CIH group (Figure 1I), whereas TC increased from 3.34 ± 0.53 mmol/L in the control group to 5.99 ± 0.21 mmol/L in the HFD group and to 7.36 ± 0.34 mmol/L in the HFD+CIH group (Figure 1J). Together, these results indicate that CIH aggravates HFD-induced metabolic dysfunction, increases liver burden, and exacerbates liver injury.

### 3.2. Multi-Omics Analyses Reveal Hepatic Molecular Reprogramming Induced by CIH Under HFD Conditions

**Figure 2:**
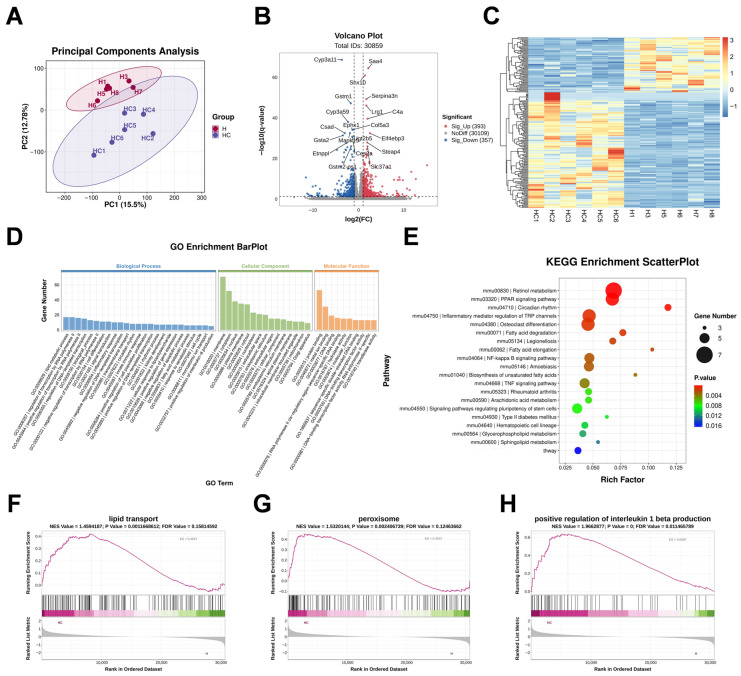
Transcriptomic profiling reveals distinct hepatic molecular reprogramming between the HFD and HFD+CIH groups. (**A**) PCA score plot of hepatic transcriptomes. (**B**) Volcano plot of DEGs (|log2 fold change| ≥ 1 and FDR < 0.05). Dashed lines indicate the thresholds used for DEG screening. Red and blue dots indicate upregulated (*n* = 130) and downregulated (*n* = 62) genes, respectively. (**C**) Hierarchical clustering heatmap of the DEGs. (**D**) GO enrichment analysis of the DEGs. (**E**) KEGG pathway enrichment analysis of the DEGs. (**F**) Representative GSEA plot of lipid transport. (**G**) Representative GSEA plot of peroxisome. (**H**) Representative GSEA plot of positive regulation of interleukin-1 beta production. Color gradients in panels F–H indicate the ranked gene list metric used for GSEA. Liver samples from *n* = 6 mice per group were used for transcriptomic analysis.

**Figure 3:**
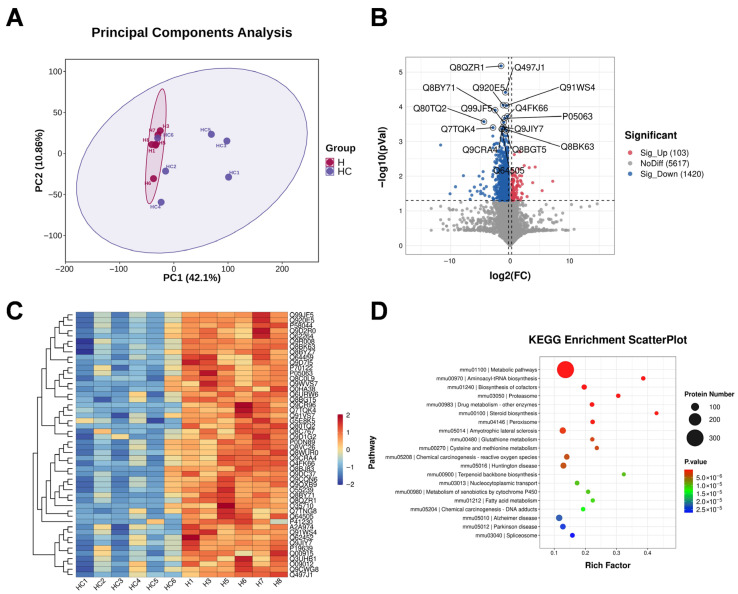
Proteomic profiling reveals distinct hepatic proteomic reprogramming between the HFD and HFD+CIH groups. (**A**) PCA score plot of hepatic proteomes. (**B**) Volcano plot of DEPs (fold change > 1.2 or < 0.83, *p* < 0.05). Dashed lines indicate the thresholds used for DEP screening. Red and blue dots indicate upregulated (*n* = 103) and downregulated (*n* = 1420) proteins, respectively. (**C**) Hierarchical clustering heatmap of the DEPs. (**D**) KEGG pathway enrichment analysis of the DEPs. Liver samples from *n* = 6 mice per group were used for proteomic analysis.

**Figure 4:**
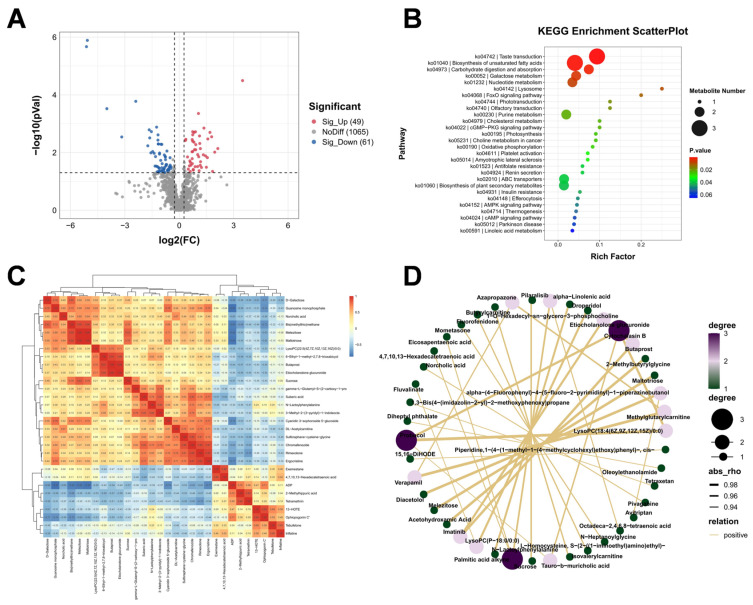
Metabolomic profiling reveals distinct hepatic metabolic reprogramming between the HFD and HFD+CIH groups. (**A**) Volcano plot of differential metabolites. Dashed lines indicate the thresholds used for differential metabolite screening. Red and blue dots indicate significantly upregulated (*n* = 49) and downregulated (*n* = 61) metabolites, respectively, whereas gray dots indicate metabolites without significant differential abundance. Differential metabolites were identified using *p* < 0.05, fold change > 1.2, and VIP ≥ 1. (**B**) KEGG pathway enrichment analysis of the differential metabolites. (**C**) Hierarchical clustering heatmap of the differential metabolites. (**D**) Correlation network of the differential metabolites. Liver samples from *n* = 6 mice per group were used for metabolomic analysis.

**Figure 5:**
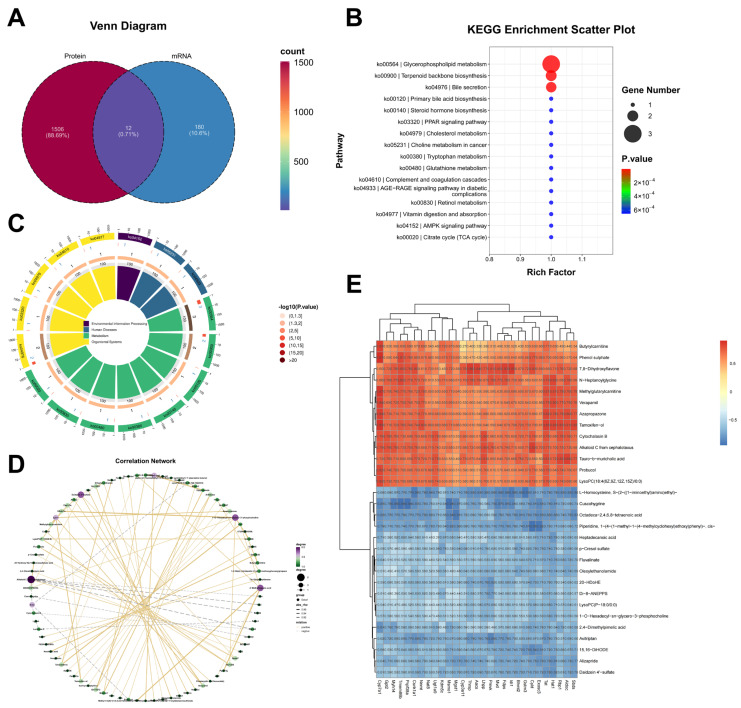
Integrated multi-omics analysis identifies convergent pathways and candidate core molecules associated with CIH-aggravated liver injury under HFD conditions. (**A**) Venn diagram showing 12 overlapping differential molecules identified at the transcriptomic and proteomic levels in the HFD+CIH versus HFD comparison. (**B**) KEGG pathway enrichment analysis of the overlapping molecules. (**C**) Circos plot showing the functional classification and pathway distribution of the overlapping molecules. (**D**) Correlation network of differential mRNAs and metabolites. (**E**) Correlation heatmap of differential proteins and metabolites. Integrated multi-omics analyses were based on liver samples from *n* = 6 mice per group.

To systematically characterize the molecular alterations induced by CIH under HFD conditions, transcriptomic, proteomic, and metabolomic profiling was performed using liver tissues from the HFD and HFD+CIH groups, followed by integrated multi-omics analysis (Figure 2, Figure 3, Figure 4 and Figure 5).

Transcriptomic analysis revealed marked hepatic transcriptional reprogramming in response to CIH. Principal component analysis showed clear separation between the HFD and HFD+CIH groups, indicating distinct global transcriptomic profiles (Figure 2A). Using the thresholds of |log2 fold change| ≥ 1 and FDR < 0.05, 192 differentially expressed genes (DEGs) were identified in the HFD+CIH group relative to the HFD group, including 130 up-regulated genes and 62 down-regulated genes (Figure 2B). Hierarchical clustering further demonstrated good intra-group consistency and clear segregation between groups (Figure 2C). GO enrichment analysis indicated that these DEGs were mainly associated with transcriptional regulation, cellular metabolic processes, and stress-related signaling (Figure 2D). KEGG enrichment analysis further highlighted pathways related to lipid and fatty acid metabolism, including retinol metabolism, the PPAR signaling pathway, fatty acid degradation, and fatty acid elongation (Figure 2E). In addition, GSEA showed coordinated alterations in pathways related to lipid metabolism, antioxidant defense, and inflammatory cytokine signaling (Figure 2F–H), suggesting that CIH induces broad transcriptional changes involving metabolic, redox, and inflammatory programs under HFD conditions.

Proteomic profiling further supported extensive molecular remodeling in the liver after CIH exposure. Principal component analysis demonstrated distinct clustering of the HFD and HFD+CIH groups at the proteomic level (Figure 3A). Differential abundance analysis identified 1523 differentially expressed proteins (DEPs), including 103 up-regulated proteins and 1420 down-regulated proteins in the HFD+CIH group relative to the HFD group (Figure 3B). Hierarchical clustering again showed clear separation between groups (Figure 3C). KEGG enrichment analysis revealed that these DEPs were mainly enriched in pathways related to lipid and fatty acid metabolism, peroxisomal function, glutathione metabolism, and xenobiotic/drug metabolism (Figure 3D). Together, these proteomic results support a broad hepatic protein abundance shift involving lipid metabolism, peroxisomal function, glutathione metabolism, and xenobiotic/drug metabolism under CIH exposure.

Untargeted metabolomic analysis identified additional metabolic disturbances associated with CIH-aggravated liver injury. A volcano plot showed widespread alterations in metabolite abundance between the HFD and HFD+CIH groups, with both significantly increased and decreased metabolites detected in the HFD+CIH group (Figure 4A). KEGG enrichment analysis indicated that these differential metabolites were mainly involved in pathways associated with lipid metabolism and energy regulation (Figure 4B). Hierarchical clustering of differential metabolites further demonstrated distinct metabolic patterns between groups (Figure 4C). Correlation network analysis revealed coordinated associations among altered metabolites, suggesting that CIH induced structured metabolic remodeling rather than isolated metabolite fluctuations (Figure 4D).

To integrate these molecular changes across omics layers, a combined multi-omics analysis was performed (Figure 5A–E). Venn analysis identified 12 overlapping molecules showing differential changes at both the transcriptomic and proteomic levels, whereas most alterations remained unique to each omics layer, indicating both reproducibility and complementarity across platforms (Figure 5A). KEGG enrichment analysis of the overlapping molecules showed predominant enrichment in pathways related to glycerophospholipid metabolism, bile secretion, primary bile acid biosynthesis, steroid hormone biosynthesis, cholesterol metabolism, retinol metabolism, and the PPAR signaling pathway (Figure 5B). A circular plot further illustrated the functional classification and pathway distribution of these overlapping molecules (Figure 5C). Correlation network analysis of differential mRNAs and metabolites demonstrated dense cross-omics associations in the HFD+CIH versus HFD comparison (Figure 5D), whereas the correlation heatmap of differential proteins and metabolites showed coordinated clustering patterns across molecular layers (Figure 5E). Collectively, these multi-omics results indicate that CIH induces coordinated hepatic molecular reprogramming under HFD conditions, with lipid metabolism, redox homeostasis, and inflammatory signaling emerging as the major affected biological programs.

### 3.3. CIH Exacerbates Hepatic Steatosis and Lipid Metabolic Reprogramming in HFD-Fed Mice

To determine whether CIH aggravates hepatic steatosis under HFD-induced metabolic stress, histological, ultrastructural, and molecular analyses were performed to evaluate lipid accumulation and lipid metabolism-related regulatory pathways.

**Figure 6:**
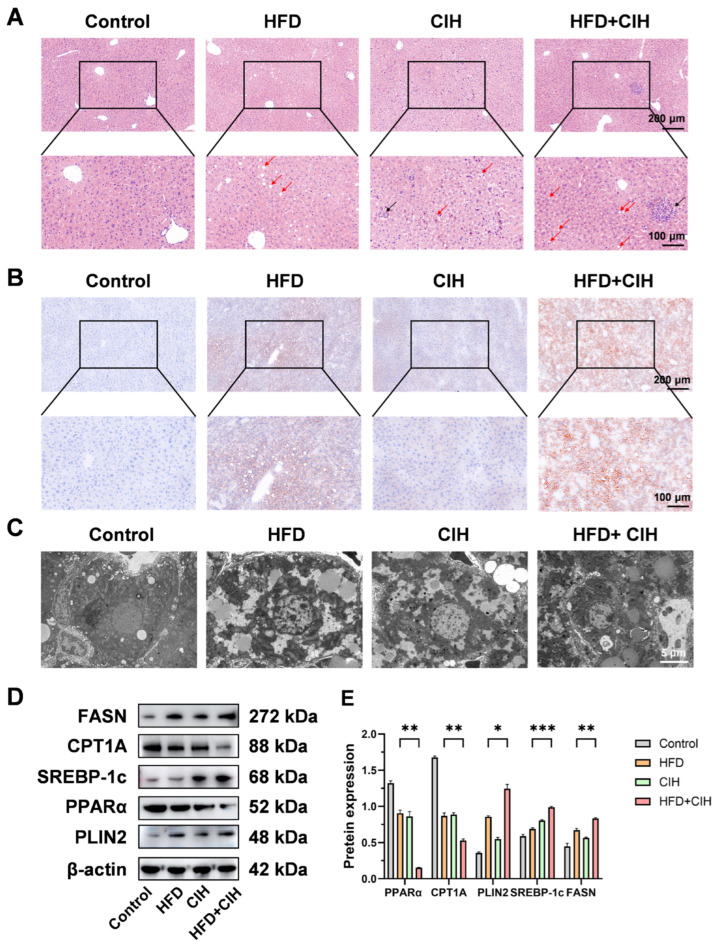
CIH aggravates hepatic steatosis and lipid metabolism-related protein dysregulation in HFD-fed mice. (**A**) Representative H&E staining images of liver sections from the Control, HFD, CIH, and HFD+CIH groups. Red arrows indicate hepatocellular vacuolar/ballooning degeneration, and black arrows indicate inflammatory cell infiltration. Upper panels, low magnification; lower panels, corresponding higher magnification views. Scale bars: 200 μm and 100 μm. (**B**) Representative Oil Red O staining images of liver sections showing neutral lipid accumulation in each group. Upper panels, low magnification; lower panels, corresponding higher magnification views. Scale bars: 200 μm and 100 μm. (**C**) Representative TEM images showing hepatocellular ultrastructural alterations in each group, with an emphasis on intracellular lipid droplet accumulation. Scale bar: 5 μm. (**D**) Representative Western blot bands of PPARα, CPT1A, PLIN2, SREBP-1c, and FASN in liver tissues from each group. (**E**) Quantitative analysis of the relative protein expression levels shown in panel D after normalization to β-actin. Data are presented as mean ± SD (*n* = 3). * *p* < 0.05, ** *p* < 0.01, *** *p* < 0.001. The original Western blot images can be found in the Supplementary Materials File S1.

Histological examination showed that HFD feeding induced evident hepatic steatotic injury, which was further aggravated by CIH. As shown in Figure 6A, liver sections from control mice displayed an intact lobular architecture, orderly hepatic cords, and no obvious inflammatory infiltration or hepatocellular swelling. In contrast, the HFD group exhibited hepatocellular vacuolar/ballooning degeneration and mild inflammatory changes, whereas the CIH group showed focal hepatocellular injury with scattered inflammatory cell infiltration. Notably, the HFD+CIH group displayed the most severe histopathological abnormalities, characterized by more extensive vacuolar/ballooning degeneration, increased inflammatory cell infiltration, and more obvious disruption of hepatic architecture. Oil Red O staining further confirmed this trend. Minimal neutral lipid deposition was observed in the control group, whereas the HFD group showed clear lipid droplet accumulation within the hepatic parenchyma. Although the CIH group showed only limited lipid deposition, the HFD+CIH group exhibited broader and denser Oil Red O-positive staining than the HFD group, indicating that CIH further promoted hepatic neutral lipid accumulation in HFD-fed mice (Figure 6B). These histological findings further support that CIH aggravates HFD-induced hepatic lipid deposition and promotes a more pronounced injurious phenotype under metabolic stress.

Ultrastructural analysis by transmission electron microscopy provided additional evidence of aggravated steatotic injury. At the ultrastructural level, hepatocytes from control mice showed relatively preserved intracellular architecture, whereas hepatocytes from the HFD group contained more lipid droplet-like structures and displayed disturbed organelle organization. Similar but milder changes were observed in the CIH group. In contrast, the HFD+CIH group showed more prominent intracellular lipid droplet accumulation and more severe subcellular disorganization than the HFD group (Figure 6C), supporting the notion that CIH aggravates HFD-induced hepatic steatosis at the ultrastructural level. Quantitative TEM analysis further showed that CIH increased lipid droplet area in HFD-fed mice (Figure S1A).

To further investigate the molecular basis underlying these phenotypic changes, key regulators involved in lipid metabolic reprogramming were evaluated. Western blot analysis showed that the fatty acid β-oxidation-related proteins PPARα and CPT1A were reduced in the HFD group compared with the control group and were further decreased in the HFD+CIH group (Figure 6D,E), indicating additional suppression of hepatic fatty acid β-oxidation under combined HFD and CIH exposure. In contrast, the lipogenesis- and lipid droplet-associated proteins SREBP-1c, FASN, and PLIN2 were increased in the HFD group and reached higher levels in the HFD+CIH group (Figure 6D,E), indicating enhanced lipid synthesis and storage in response to CIH superimposition. These protein-level changes were further supported by RT-qPCR analysis, which showed reduced expression of the fatty acid β-oxidation-related genes *Ppara* and *Cpt1a* and increased expression of the lipid synthesis/storage-related genes *Srebf1*, *Fasn*, and *Plin2* in the HFD+CIH group (Figure S1B,C). Collectively, these results indicate that CIH exacerbates hepatic steatosis in HFD-fed mice by further suppressing fatty acid β-oxidation while enhancing lipid synthesis and lipid droplet storage, thereby driving hepatic lipid metabolic reprogramming.

### 3.4. CIH Impairs Antioxidant Defense and Promotes Oxidative Stress-Related Liver Injury

**Figure 7:**
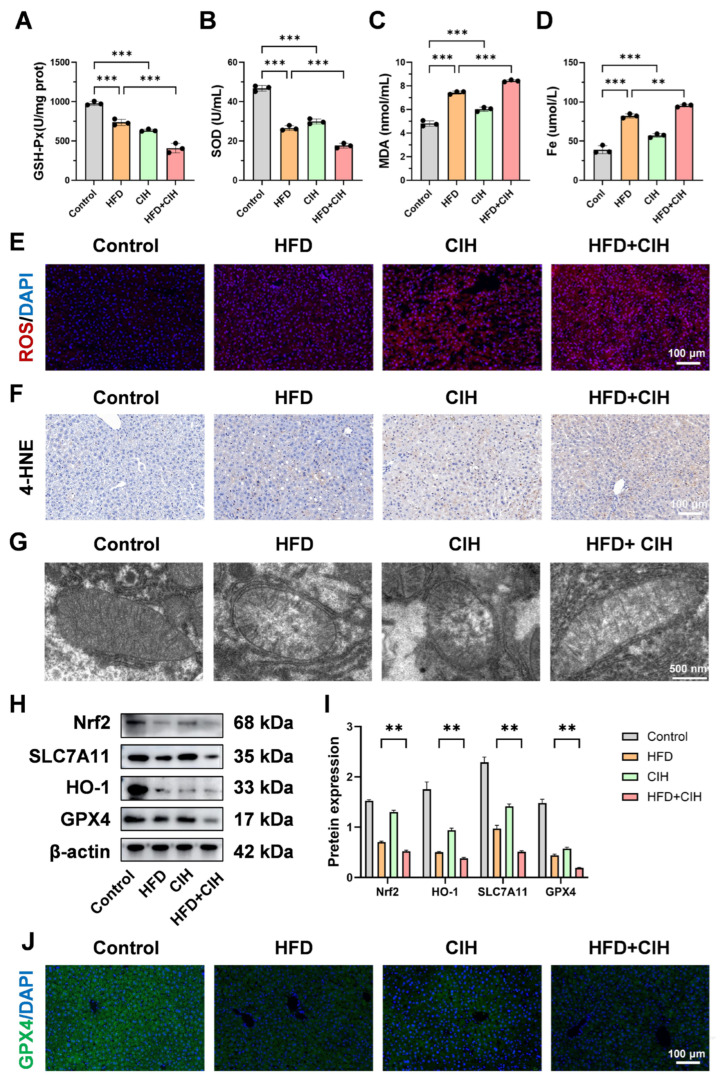
CIH impairs the Nrf2–GPX4 antioxidant/lipid peroxidation defense axis in HFD-fed mice. (**A**–**D**) Hepatic oxidative stress- and lipid peroxidation-related biochemical parameters, including GSH-Px activity, SOD activity, MDA level, and Fe content. (**E**) Representative ROS fluorescence staining images of liver sections from the Control, HFD, CIH, and HFD+CIH groups. (**F**) Representative 4-HNE immunohistochemical staining images of liver sections. (**G**) Representative high-magnification TEM images showing mitochondrial morphology in liver tissues from each group. Scale bar: 500 nm. (**H**) Representative Western blot bands of Nrf2, SLC7A11, HO-1, and GPX4, with β-actin as the loading control. (**I**) Quantitative analysis of the relative protein expression levels shown in panel (**H**). (**J**) Representative GPX4 immunofluorescence staining images of liver sections. Data are presented as mean ± SD (*n* = 3). ** *p* < 0.01, *** *p* < 0.001. The original Western blot images can be found in the Supplementary Materials File S1.

To determine whether CIH aggravates oxidative stress-related liver injury under HFD-induced metabolic stress, biochemical, histological, and molecular analyses were performed to evaluate oxidative stress, lipid peroxidation, and antioxidant defense status (Figure 7 and Figure S2).

Biochemical analyses showed that hepatic antioxidant capacity was impaired by HFD feeding and further deteriorated after CIH exposure. As shown in Figure 7A,B, GSH-Px and SOD activities were reduced in the HFD group compared with the control group and were further decreased in the HFD+CIH group, indicating a progressive decline in hepatic antioxidant defense. In contrast, MDA levels were elevated by HFD feeding and increased further after CIH exposure (Figure 7C), suggesting enhanced lipid peroxidation under combined HFD and CIH conditions. In addition, hepatic Fe content was increased in the HFD+CIH group relative to the HFD group (Figure 7D), further supporting aggravated oxidative injury in the presence of CIH. Together, these findings indicate that CIH further weakens hepatic antioxidant capacity and aggravates oxidative stress under HFD conditions.

Histological analyses further confirmed enhanced oxidative stress and lipid peroxidation at the tissue level. ROS fluorescence staining showed that hepatic ROS signals were increased in the HFD group compared with the control group and were further enhanced in the HFD+CIH group (Figure 7E). Likewise, 4-HNE immunohistochemical staining revealed stronger positive signals in the HFD group and an additional increase in the HFD+CIH group (Figure 7F), indicating increased accumulation of lipid peroxidation end products under CIH superimposition. Quantitative analyses of ROS fluorescence intensity and 4-HNE-positive area in Figure S2A,B further supported these observations. High-magnification TEM imaging further showed that mitochondria in the HFD+CIH group displayed more pronounced ultrastructural abnormalities, characterized by less clearly organized cristae structures and altered mitochondrial morphology, compared with those in the HFD group (Figure 7G).

To further investigate the molecular basis underlying these changes, key components of the Nrf2-related antioxidant defense pathway were examined. Western blot analysis showed that the protein levels of Nrf2, HO-1, SLC7A11, and GPX4 were reduced in the HFD group and were further decreased in the HFD+CIH group (Figure 7H,I), indicating progressive suppression of the Nrf2-centered antioxidant defense axis. Consistently, GPX4 immunofluorescence in liver sections was weaker in the HFD+CIH group than in the HFD group (Figure 7J), and quantitative analysis in Figure S2C confirmed this reduction. At the transcriptional level, RT-qPCR further showed that Nfe2l2, Hmox1, Slc7a11, and Gpx4 were all significantly downregulated in the HFD+CIH group relative to the HFD group (Figure S2D), supporting coordinated impairment of antioxidant defense at both the mRNA and protein levels.

Collectively, these results indicate that CIH aggravates oxidative stress-related liver injury in HFD-fed mice by suppressing the Nrf2–SLC7A11/HO-1/GPX4 antioxidant defense axis, enhancing ROS accumulation, and promoting lipid peroxidation.

### 3.5. CIH Enhances Hepatic Inflammation and Activates the NF-κB/NLRP3 Signaling Axis

**Figure 8:**
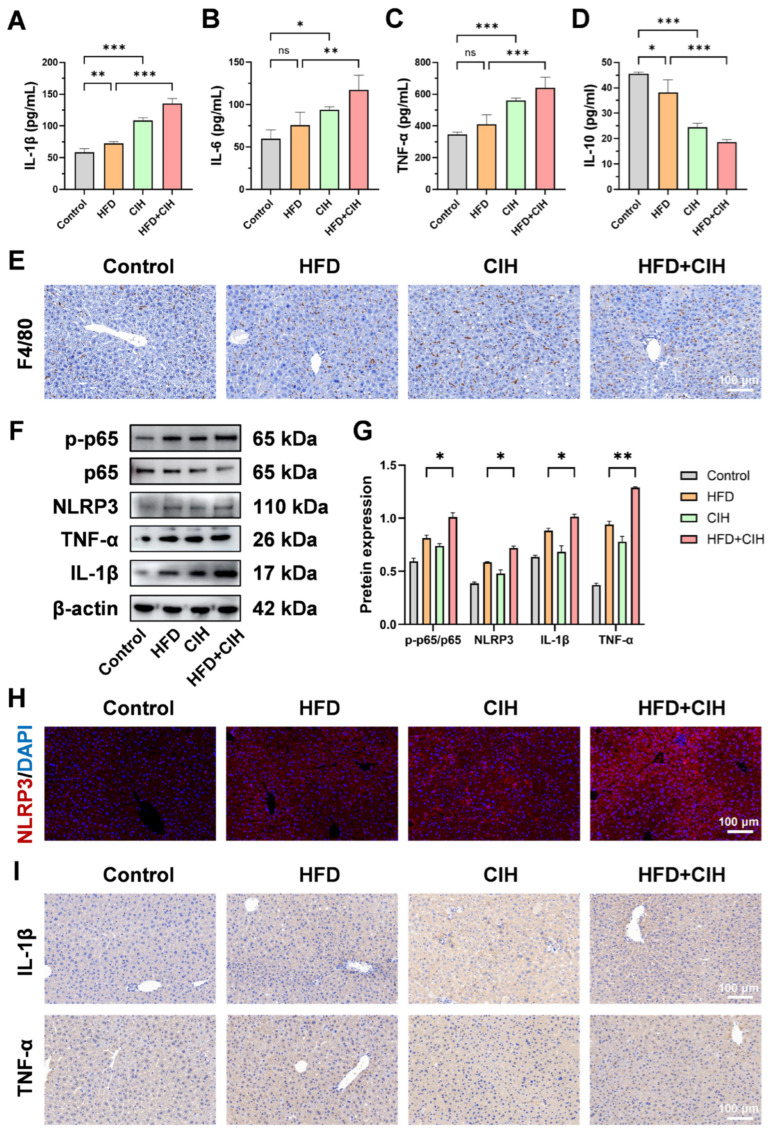
CIH activates the NF-κB/NLRP3 inflammatory axis and aggravates hepatic inflammatory responses in HFD-fed mice. (**A**–**D**) Serum levels of inflammatory cytokines, including IL-1β (**A**), IL-6 (**B**), TNF-α (**C**), and IL-10 (**D**). (**E**) Representative immunohistochemical staining images of F4/80 in liver sections from the Control, HFD, CIH, and HFD+CIH groups. Scale bar: 100 μm. (**F**) Representative Western blot bands of p-p65, p65, NLRP3, TNF-α, and IL-1β, with β-actin as the loading control. (**G**) Quantitative analysis of the p-p65/p65 ratio and the relative protein expression levels of NLRP3, IL-1β, and TNF-α shown in panel (**F**). (**H**) Representative NLRP3 immunofluorescence staining images of liver sections from each group. Scale bar: 100 μm. (**I**) Representative immunohistochemical staining images of IL-1β and TNF-α in liver sections from each group. Scale bar: 100 μm. Data are presented as mean ± SD (*n* = 3). * *p* < 0.05, ** *p* < 0.01, *** *p* < 0.001. The original Western blot images can be found in the Supplementary Materials File S1.

To determine whether CIH aggravates hepatic inflammatory injury under HFD-induced metabolic stress, systemic inflammatory cytokines, hepatic macrophage infiltration, and NF-κB/NLRP3 pathway activation were evaluated using serum ELISA, histological staining, Western blotting, and immunofluorescence analyses (Figure 8).

Serum cytokine analysis showed that HFD feeding induced a pro-inflammatory shift that was further amplified by CIH exposure. As shown in Figure 8A–D, serum IL-1β, IL-6, and TNF-α levels were increased in the HFD group compared with the control group and were further elevated in the HFD+CIH group, whereas the anti-inflammatory cytokine IL-10 showed the opposite trend and was further reduced after CIH superimposition. These findings indicate that CIH aggravates systemic inflammatory responses in HFD-fed mice.

Histological evidence further supported enhanced hepatic inflammation. F4/80 immunohistochemical staining showed weak macrophage signals in the control group, increased staining in the HFD and CIH groups, and the strongest staining in the HFD+CIH group (Figure 8E). These findings indicate enhanced hepatic macrophage infiltration after CIH superimposition.

To further investigate the molecular basis underlying these inflammatory changes, the NF-κB/NLRP3 signaling axis was examined. Western blot analysis showed that the p-p65/p65 ratio was increased in the HFD+CIH group, accompanied by elevated protein expression levels of NLRP3, IL-1β, and TNF-α (Figure 8F,G), supporting activation of the NF-κB/NLRP3 inflammatory axis. In parallel, NLRP3 immunofluorescence staining showed stronger tissue-level signals in the HFD+CIH group than in the HFD group (Figure 8H). In addition, immunohistochemical staining demonstrated increased hepatic IL-1β and TNF-α signals in the HFD+CIH group (Figure 8I), further supporting enhanced local inflammatory responses after CIH exposure. These observations further support activation of hepatic NF-κB/NLRP3 signaling by CIH under HFD conditions.

Collectively, these results indicate that CIH aggravates hepatic inflammatory injury in HFD-fed mice by enhancing macrophage infiltration and activating the NF-κB/NLRP3 signaling axis.

## 4. Discussion

In the present study, we demonstrated that CIH aggravates HFD-induced MASLD-like liver injury at phenotypic, histological, ultrastructural, and molecular levels. CIH further increased body fat accumulation, liver burden, serum transaminases, and dyslipidemia in HFD-fed mice, and exacerbated hepatic steatosis, oxidative stress, lipid peroxidation, and inflammatory responses. Multi-omics analyses and targeted validation further indicated that these alterations converged on three interconnected axes: dysregulation of lipid metabolic homeostasis, impairment of antioxidant defense, and activation of inflammatory signaling. Collectively, these findings support a multifactorial model in which CIH functions as an additional hypoxic stressor that aggravates MASLD-like liver injury under conditions of pre-existing metabolic stress [11,19,20].

This overall framework is biologically plausible and consistent with the current understanding of MASLD as a multifactorial disease driven by the interaction of metabolic overload, oxidative injury, and inflammatory amplification rather than by a single pathogenic insult [20,21]. In this setting, HFD provides a basal metabolic burden, whereas CIH appears to lower the threshold for transition from steatosis toward a more injurious phenotype. This interpretation is also in line with growing clinical evidence indicating that OSA is associated not only with hepatic steatosis, but also with more severe liver injury and fibrosis-related phenotypes, even after adjustment for shared metabolic risk factors [22,23,24]. Thus, our study extends the epidemiologic association between OSA-related hypoxic stress and fatty liver disease by providing controlled experimental evidence that CIH can aggravate liver injury in the setting of metabolic overload.

A major finding of the present study is that CIH exacerbated hepatic steatosis and was accompanied by coordinated alterations in lipid metabolism-related pathways. Histology, Oil Red O staining, and TEM consistently showed more severe lipid accumulation and structural disruption after CIH superimposition, whereas transcriptomic, proteomic, and validation analyses showed suppression of fatty acid β-oxidation together with enhancement of lipogenesis and lipid droplet storage. This pattern is mechanistically meaningful because PPARα is a key regulator of hepatic fatty acid oxidation, CPT1A controls mitochondrial fatty acid entry, SREBP-1c is a central transcriptional driver of de novo lipogenesis, FASN mediates fatty acid synthesis, and PLIN2 promotes lipid droplet stabilization and retention [25,26,27,28,29]. Therefore, the simultaneous downregulation of PPARα/CPT1A and upregulation of SREBP-1c/FASN/PLIN2 observed in our model suggests that CIH does not merely increase lipid delivery to the liver, but actively reprograms hepatic lipid handling toward storage and away from oxidation.

This lipid metabolic phenotype is consistent with previous work showing that hypoxia can aggravate fatty liver disease through suppression of PPARα-associated fatty acid oxidation and enhancement of steatogenic signaling [30,31]. It also fits with the broader concept that CIH may promote adipose lipolysis, increase free fatty acid influx into the liver, and thereby enhance lipotoxic stress in metabolically susceptible hosts [32,33]. Importantly, our study adds to this literature in two respects. First, the multi-omics results indicate that lipid metabolism is not one of many equally altered pathways, but a central convergent module across transcriptomic, proteomic, and metabolomic layers. Second, the validation data show that the CIH effect is translated into a stable phenotypic shift in the balance between lipid oxidation and lipid storage. From a disease-progression perspective, this shift is highly relevant because persistent intracellular lipid retention provides the substrate for subsequent oxidative injury and inflammatory activation [34].

Another key contribution of this study is the demonstration that CIH markedly impaired antioxidant defense and enhanced oxidative stress and lipid peroxidation in HFD-fed mice. The HFD+CIH group exhibited lower SOD and GSH-Px activities, higher ROS and MDA levels, increased 4-HNE staining, and reduced expression of Nrf2, HO-1, SLC7A11, and GPX4. These findings are important because oxidative stress is widely recognized as a major driver of progression from simple steatosis to steatohepatitis-like injury [35]. Although hypoxia itself reduces oxygen availability, CIH is characterized by repeated hypoxia–reoxygenation cycles rather than sustained oxygen deprivation. During reoxygenation, ROS generation may be enhanced in a manner resembling ischemia–reperfusion-like stress [15,36]. In the setting of HFD-induced lipid overload, increased oxidizable lipid substrates and weakened antioxidant defenses may together render hepatocytes more susceptible to lipid peroxidation. Particularly noteworthy is the suppression of the Nrf2-centered antioxidant defense system. Nrf2 is a master regulator of cellular antioxidant responses, whereas SLC7A11 and GPX4 are critical components linking glutathione metabolism to the detoxification of lipid peroxides [37]. HO-1 is another major stress-responsive target involved in adaptive antioxidant defense [38]. The coordinated downregulation of these molecules suggests that CIH not only increases oxidant burden but simultaneously weakens hepatocellular defenses against lipid peroxidation. In the setting of HFD-induced lipid overload, where abundant lipid substrates are already available for oxidative chain reactions, this dual effect is likely particularly damaging. Therefore, rather than simply increasing oxidative stress in a general sense, CIH appears to drive a pro-peroxidative state by disrupting a key protective system that normally limits membrane lipid damage.

The inflammatory data further reinforce this mechanistic framework. In the HFD+CIH group, circulating IL-1β, IL-6, and TNF-α were elevated, IL-10 was reduced, F4/80-positive macrophage infiltration was enhanced, and hepatic NF-κB/NLRP3 signaling was activated. These observations are biologically coherent because lipotoxicity, oxidative stress, and hepatocellular injury are all capable of triggering inflammatory pathway activation in MASLD [39]. NF-κB functions as a major transcriptional hub linking stress signals to cytokine production, whereas the NLRP3 inflammasome promotes maturation of IL-1β and amplifies inflammatory liver injury [40,41]. Accordingly, the increased p-p65/p65 ratio and enhanced expression of NLRP3, IL-1β, and TNF-α in our study suggest that CIH does not merely induce a nonspecific inflammatory response, but actively drives a pro-inflammatory signaling program in the metabolically stressed liver.

Importantly, the inflammatory changes observed here should not be viewed in isolation from the lipid and oxidative alterations described above. Rather, our findings support a coupled mechanism in which lipid metabolic reprogramming increases intracellular lipid burden, oxidative injury promotes lipid peroxidation and stress signaling, and inflammatory activation further exacerbates hepatocellular damage and immune cell recruitment. Such coupling is highly relevant to MASLD pathogenesis because it is the interaction among these processes, rather than any single pathway alone, that determines progression toward a more severe phenotype [42,43]. This integrative view is also supported by our multi-omics results, in which lipid metabolism, redox homeostasis, and inflammatory signaling repeatedly emerged as convergent pathway modules across multiple analytical layers. Thus, one conceptual advance of the present study is that it defines a reproducible systems-level mechanism through which CIH aggravates HFD-induced liver injury.

The multi-omics component is another strength of this study. Most previous experimental studies addressing CIH-related liver injury have focused on selected candidate pathways or limited molecular markers [44,45]. In contrast, the integrated transcriptomic, proteomic, and metabolomic analyses performed here allowed us to identify not only individual molecules but also higher-order pathway convergence. This is particularly important in a complex disease such as MASLD, in which transcriptional changes, protein abundance shifts, and terminal metabolic outputs are often only partially overlapping. The fact that lipid-associated, redox-related, and inflammatory modules were repeatedly highlighted across omics layers increases confidence that these are not incidental findings. At the same time, the incomplete overlap among omics platforms also indicates that CIH-related liver injury involves multilevel regulation and cannot be fully explained by a single layer of analysis. In this sense, the multi-omics design strengthens the mechanistic credibility of our conclusions and provides a broader framework than traditional single-pathway studies [46].

It should also be noted that not all published studies have reported identical effects of intermittent hypoxia on fatty liver phenotypes. Some experimental studies have yielded apparently inconsistent observations, likely due to differences in oxygen nadir, cycle frequency, exposure duration, dietary background, animal strain, and baseline metabolic state [47,48]. In our view, such heterogeneity does not weaken the present findings, but instead highlights the importance of modeling CIH within a clinically relevant metabolic context. Our study specifically examined CIH superimposed on HFD-induced metabolic stress, which more closely reflects the real-world coexistence of OSA with obesity and metabolic dysfunction. Under these conditions, the aggravating effect of CIH on steatotic liver injury was robust across phenotypic, histological, ultrastructural, and molecular readouts.

From a translational perspective, the present findings may have at least two implications. First, they reinforce the concept that OSA-related hypoxic stress should not be regarded as a simple comorbidity in patients with fatty liver disease, but rather as a potential disease modifier that accelerates progression by reshaping the hepatic metabolic and inflammatory microenvironment [49]. Second, the pathways identified here—particularly the PPARα/CPT1A axis, the Nrf2–SLC7A11/HO-1/GPX4 axis, and the NF-κB/NLRP3 axis—may represent rational targets for mechanism-guided intervention in CIH-related MASLD. This is especially relevant given that the hepatic benefits of OSA treatment itself remain uncertain and may be heterogeneous across studies [50]. Therefore, combined strategies targeting both nocturnal hypoxic stress and downstream liver injury pathways may ultimately prove more effective than focusing on either component alone.

Several limitations should also be considered. First, although our model reproduced key features of CIH-superimposed HFD-induced liver injury, it cannot fully capture the complexity of human OSA, which also involves sleep fragmentation, neurohumoral alterations, and substantial comorbidity heterogeneity [51]. The use of male C57BL/6 mice under a defined HFD and CIH exposure paradigm may also limit generalizability to heterogeneous human MASLD populations, and future validation in clinical cohorts or patient-derived samples will be needed. Second, while our multi-omics analyses and validation experiments identified convergent pathways and candidate core molecules, the current study remains primarily mechanistic-descriptive; direct gain- or loss-of-function experiments targeting specific nodes such as PPARα, GPX4, or NLRP3 were not performed. Third, we focused mainly on the transition toward a steatotic and inflammatory phenotype and did not assess fibrosis or longer-term outcomes, which are clinically important in OSA-associated liver disease progression [52]. Finally, although the integrated omics design is a major strength, not every molecular alteration observed can be interpreted as causal; some may represent adaptive or secondary responses. These issues should be addressed in future studies through targeted intervention experiments, longer-term exposure paradigms, and translational validation in human cohorts.

**Figure 9:**
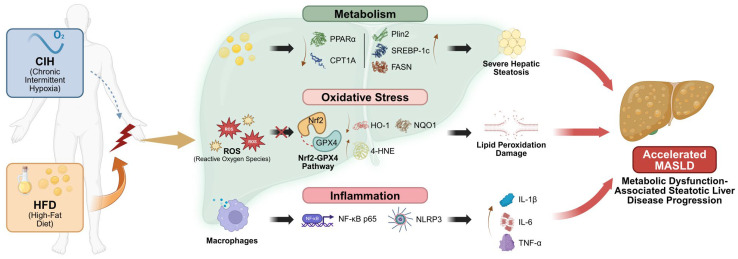
Proposed schematic model of the mechanisms by which CIH accelerates MASLD progression under HFD conditions.

In summary, our study supports a model in which CIH accelerates HFD-induced MASLD progression through coordinated lipid metabolic reprogramming, failure of antioxidant and anti-lipid peroxidation defense, and activation of inflammatory signaling. Rather than acting through a single dominant pathway, CIH appears to amplify the reciprocal interaction among metabolic overload, oxidative injury, and inflammation, thereby driving a more injurious hepatic state. Based on the phenotypic, multi-omics, and validation data, a schematic summary of the proposed mechanism is presented in Figure 9. This integrative framework may help explain why OSA is increasingly linked to more severe liver disease and may also provide a mechanistic basis for future studies aimed at identifying therapeutic strategies for CIH-related MASLD.

## 5. Conclusions

In conclusion, the present study demonstrates that chronic intermittent hypoxia markedly aggravates HFD-induced MASLD-like liver injury. By integrating phenotypic, histological, ultrastructural, transcriptomic, proteomic, metabolomic, and targeted validation data, we show that CIH promotes disease progression through coordinated disruption of three interconnected processes: lipid metabolic reprogramming, impairment of antioxidant defense with enhanced lipid peroxidation, and activation of pro-inflammatory signaling. Mechanistically, these alterations were characterized by suppression of the PPARα/CPT1A axis, inhibition of the Nrf2–SLC7A11/HO-1/GPX4 antioxidant system, and activation of the NF-κB/NLRP3 inflammatory pathway. These findings provide systems-level evidence that CIH may function as an active disease-modifying factor that exacerbates MASLD-like liver injury under conditions of metabolic overload. Collectively, this study advances the mechanistic understanding of OSA-related liver injury and highlights several biologically plausible pathways that may serve as potential targets for mechanism-guided intervention in CIH-related MASLD.

## Data Availability

The data presented in this study are available from the corresponding author upon reasonable request. The omics-related datasets and enrichment results are provided in Supplementary Tables S2–S9.

## Supplementary Materials

The following supporting information can be downloaded at: https://www.mdpi.com/article/10.3390/biom16050751/s1, Table S1: Primer sequences used for RT-qPCR; Table S2: Differentially expressed genes in the HFD+CIH versus HFD comparison; Table S3: GO enrichment results of differentially expressed genes; Table S4: KEGG enrichment results of differentially expressed genes; Table S5: GO-based transcriptomic GSEA results; Table S6: Differentially expressed proteins in the HFD+CIH versus HFD comparison; Table S7: KEGG enrichment results of differentially expressed proteins; Table S8: Differential metabolites in the HFD+CIH versus HFD comparison; Table S9: KEGG enrichment results of differential metabolites; Figure S1: TEM-based quantification of lipid droplet accumulation and RT-qPCR analysis of lipid metabolism-related genes in the livers of mice from the Control, HFD, CIH, and HFD+CIH groups; Figure S2: Quantitative analyses of ROS fluorescence intensity, 4-HNE-positive area, GPX4 immunofluorescence intensity, and RT-qPCR validation of *Nfe2l2*, *Hmox1*, *Slc7a11*, and *Gpx4* in HFD+CIH mice; Figure S3: Quantitative analyses of F4/80-positive area, NLRP3 immunofluorescence intensity, IL-1β-positive area, TNF-α-positive area, and RT-qPCR validation of *Nlrp3*, *Il1b*, *Il6*, *Tnf*, and *Ccl2* in HFD+CIH mice; File S1: The original Western blot images.

## Abbreviations

The following abbreviations are used in this manuscript:

CIH	chronic intermittent hypoxia
HFD	high-fat diet
MASLD	metabolic dysfunction-associated steatotic liver disease
OSA	obstructive sleep apnea
ROS	reactive oxygen species
TEM	transmission electron microscopy
