# Supplementary material for: Chronic Intermittent Hypoxia Exacerbates High-Fat Diet-Induced MASLD Through Lipid Metabolic Reprogramming, Impaired Antioxidant Defense, and NF-κB/NLRP3 Activation
Source: Biomolecules. 2026 May 20;16(5):751. doi: 10.3390/biom16050751 (PMC13204061; doi:10.3390/biom16050751)
Supplement: Supplementary file 1 [file biomolecules-16-00751-s001.zip › Supplementary figures.pdf]

Supplementary Materials

Chronic Intermittent Hypoxia Exacerbates High-Fat Diet-Induced MASLD through Lipid Metabolic Reprogramming, Impaired Antioxidant Defense, and NF-κB/NLRP3 Activation

Lisong Ye[1,2], Jihang Le[1], Xiaofei Lei[1], Fujie Song[1,2], YiChan Guo[2], Jun Gao[2]*, Yuehua Liu[1,2]*

[1] Department of Orthodontics, Shanghai Stomatological Hospital & School of Stomatology, Fudan University, Shanghai, China; lsye23@m.fudan.edu.cn (L.Y.); 22301010025@m.fudan.edu.cn (J.L.); 22301010073@m.fudan.edu.cn (X.L.);
[2] Shanghai Key Laboratory of Craniomaxillofacial Development and Diseases, Shanghai, China;
jef0512@foxmail.com (F.S.); 22301010027@m.fudan.edu.cn (Y.G.)
* Correspondence: liuyuehua@fudan.edu.cn (Y.L.); gaojun_kq@fudan.edu.cn (J.G.)

Supplementary Tables S1–S9 are provided in the supplementary ZIP file as separate Excel files:

Table S1. Primer sequences used for RT-qPCR;
Table S2. Differentially expressed genes in the HFD+CIH versus HFD comparison;
Table S3. GO enrichment results of differentially expressed genes;
Table S4. KEGG enrichment results of differentially expressed genes;
Table S5. GO-based transcriptomic GSEA results;
Table S6. Differentially expressed proteins in the HFD+CIH versus HFD comparison;
Table S7. KEGG enrichment results of differentially expressed proteins;
Table S8. Differential metabolites in the HFD+CIH versus HFD comparison;
Table S9. KEGG enrichment results of differential metabolites.

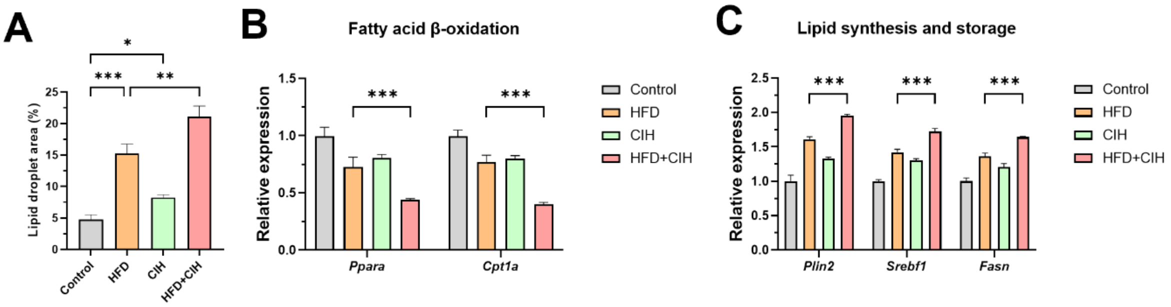

Figure S1. (A) Quantification of lipid droplet area based on TEM images. Lipid droplet accumulation was expressed as lipid droplet area percentage. (B) Relative mRNA expression levels of fatty acid β-oxidation-related genes, including *Ppara* and *Cpt1a*. (C) Relative mRNA expression levels of lipid synthesis- and lipid storage-related genes, including *Plin2*, *Srebf1*, and *Fasn*. Data are presented as mean ± SD ($n = 3$). Statistical significance for the indicated comparisons was determined by one-way ANOVA followed by Tukey's multiple-comparison test. *$p < 0.05$, **$p < 0.01$, ***$p < 0.001$.

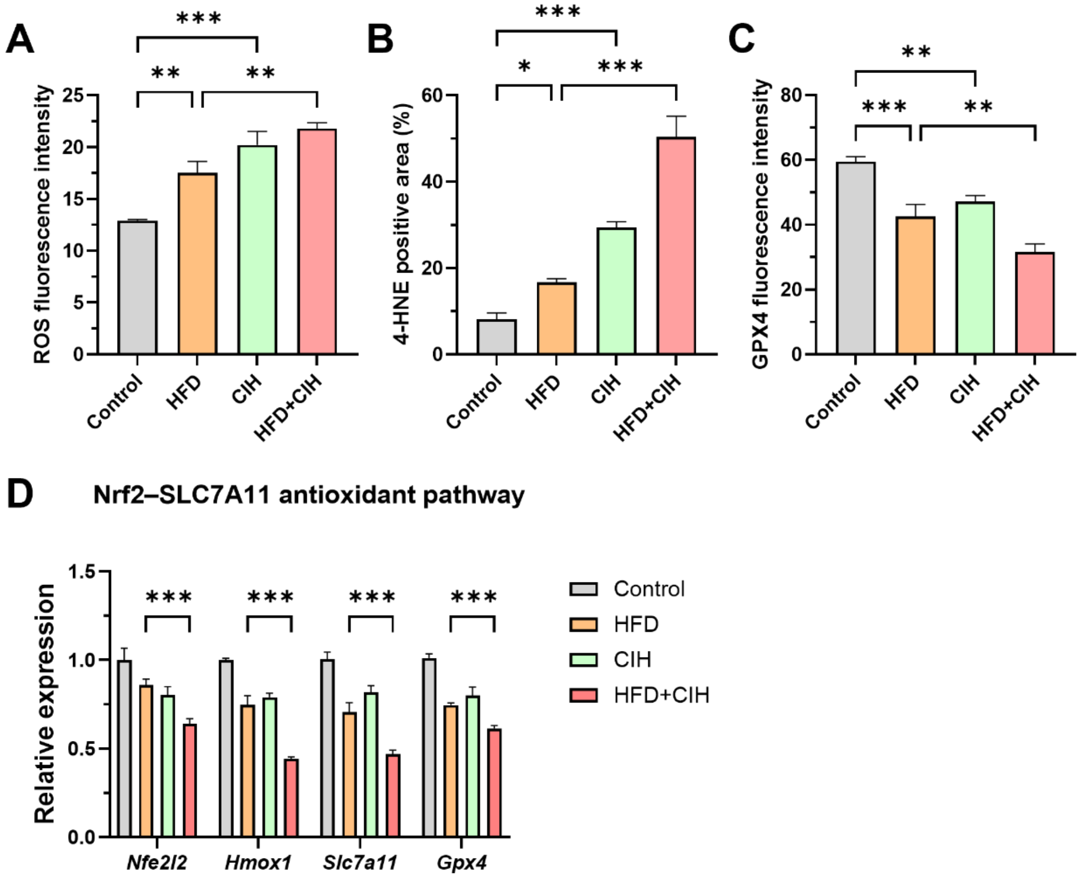

Figure S2. (A) Quantification of ROS fluorescence intensity in liver sections. (B) Quantification of 4-HNE-positive area in liver sections. (C) Quantification of GPX4 immunofluorescence intensity in liver sections. (D) Relative mRNA expression levels of *Nfe2l2*, *Hmox1*, *Slc7a11*, and *Gpx4* in liver tissues. Data are presented as mean ± SD (n = 3). Statistical significance for the indicated comparisons was determined by one-way ANOVA followed by Tukey's multiple-comparison test. $*p < 0.05$, $**p < 0.01$, $***p < 0.001$.

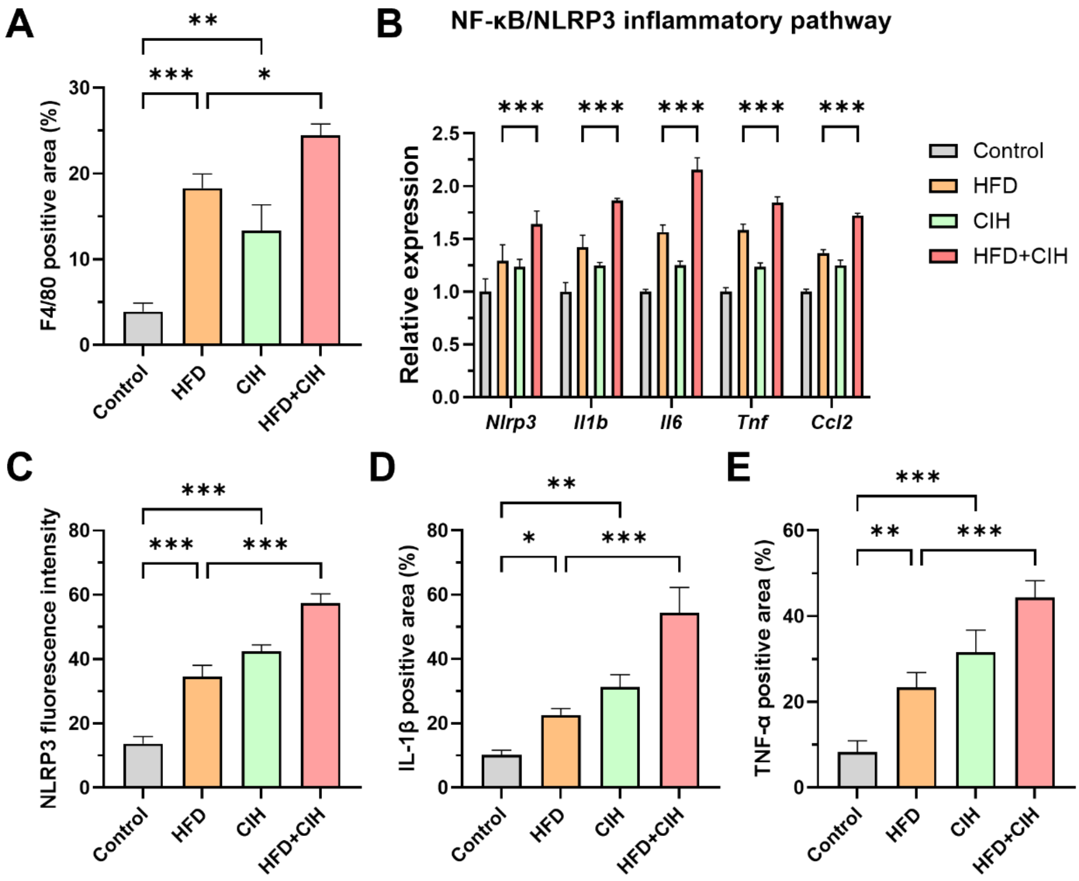

Figure S3. (A) Quantification of F4/80-positive area in liver sections. (B) Relative mRNA expression levels of *Nlrp3*, *Il1b*, *Il6*, *Tnf*, and *Ccl2* in liver tissues. (C) Quantification of NLRP3 immunofluorescence intensity in liver sections. (D) Quantification of IL-1β-positive area in liver sections. (E) Quantification of TNF-α-positive area in liver sections. Data are presented as mean ± SD (n = 3). Statistical significance for the indicated comparisons was determined by one-way ANOVA followed by Tukey's multiple-comparison test. *$p < 0.05$, **$p < 0.01$, ***$p < 0.001$.
