# Supplementary figures and images for: Chronic Intermittent Hypoxia Exacerbates High-Fat Diet-Induced MASLD Through Lipid Metabolic Reprogramming, Impaired Antioxidant Defense, and NF-κB/NLRP3 Activation
Source: Biomolecules. 2026 May 20;16(5):751. doi: 10.3390/biom16050751 (PMC13204061; doi:10.3390/biom16050751)

Figure 6D

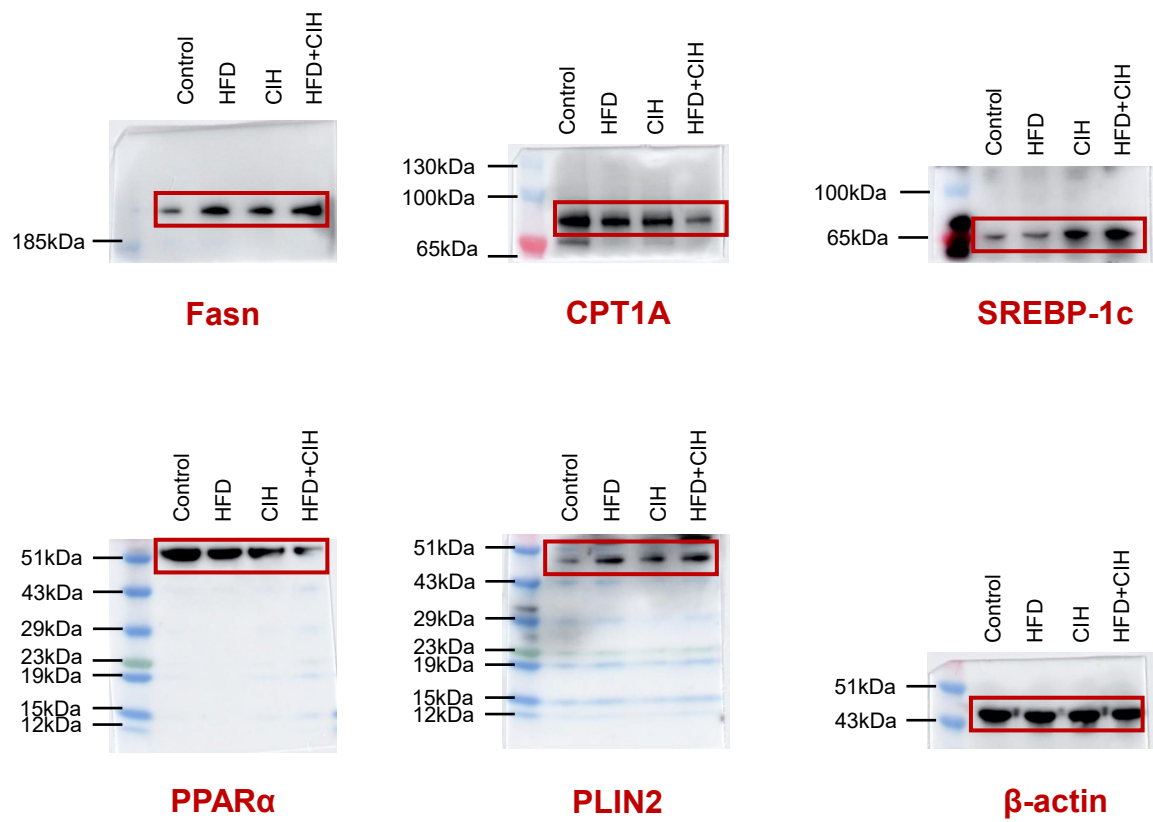

Figure 7H

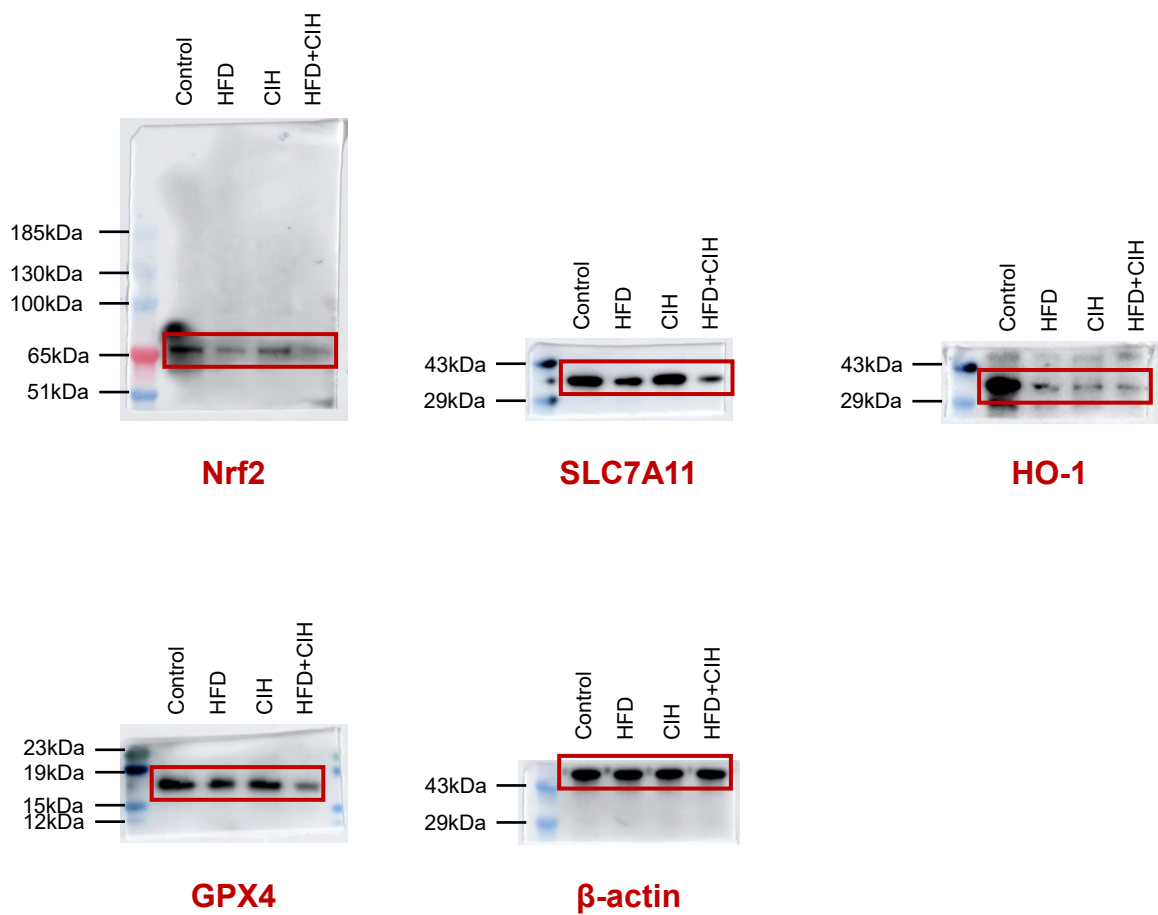

**Figure 8F**

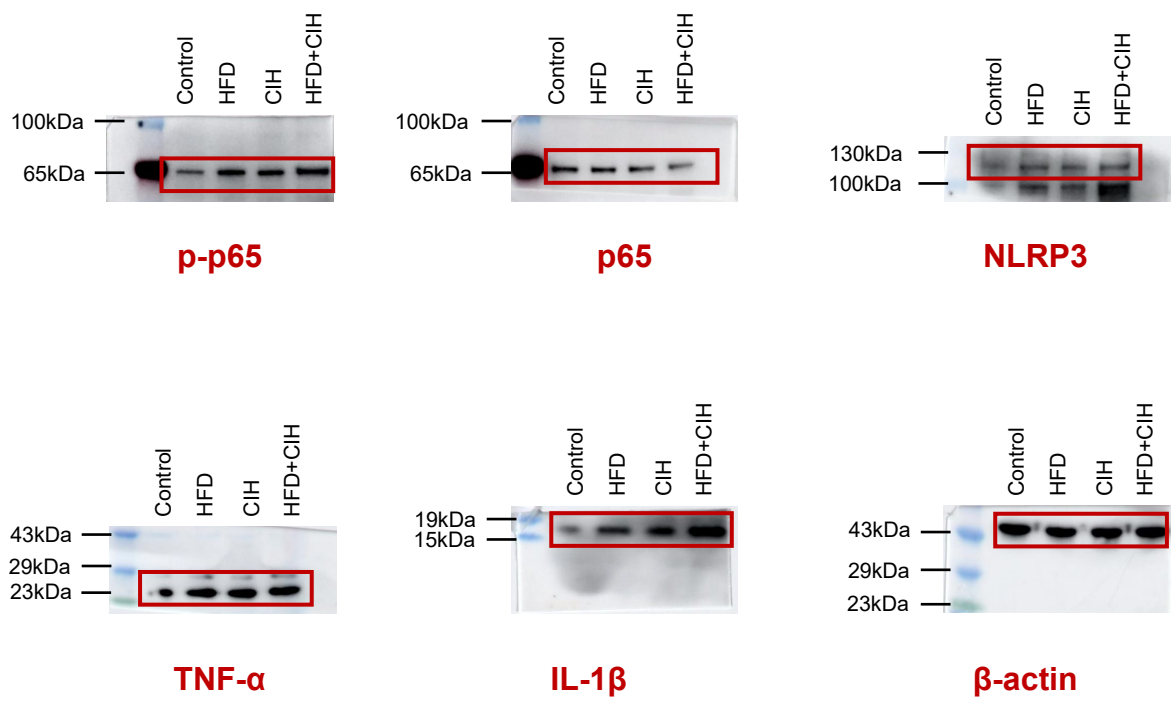

Supplement: Supplementary file 1 [file biomolecules-16-00751-s001.zip › File S1 The orginal Western blot images.pdf]
